# Supplementary material for: International Survey of Specialist Fetal Alcohol Spectrum Disorder Diagnostic Clinics: Comparison of Diagnostic Approach and Considerations Regarding the Potential for Unification
Source: Int J Environ Res Public Health. 2022 Nov 25;19(23):15663. doi: 10.3390/ijerph192315663 (PMC9737886; doi:10.3390/ijerph192315663)
Supplement: Supplementary file 1 [file ijerph-19-15663-s001.zip › ijerph-1960844-supplementary.pdf]

Diagnostic Clinic Survey

📖 Codebook

📖 Data Dictionary Codebook

08-08-2019 10:23am

#	Variable / Field Name	Field Label<br>*Field Note*	Field Attributes (Field Type, Validation, Choices, Calculations, etc.)
Instrument: **Diagnostic Clinic Survey** (diagnostic_clinic_survey)			
1	record_id	Record ID	text
2	consent	By selecting the AGREE button below, I confirm that I have read and understood the information package. I would like to give my consent and participate in the survey.	radio, Required<br>1 Agree (continue to survey)<br>2 No, I would not like to participate.<br>Custom alignment: LH
3	clinic_name<br>Show the field ONLY if:<br>[consent] = '1'	What is the name of your clinic?	notes
4	client_agerange<br>Show the field ONLY if:<br>[consent] = '1'	What is the age range of clients seen in your clinic? Select all that apply.	checkbox<br>1 client_agerange___1 0-5<br>2 client_agerange___2 6-12<br>3 client_agerange___3 13-17<br>4 client_agerange___4 18+
5	demo_setting<br>Show the field ONLY if:<br>[consent] = '1'	What type of clinic is it?	checkbox<br>1 demo_setting___1 Hospital<br>2 demo_setting___2 Community agency<br>3 demo_setting___3 University<br>4 demo_setting___4 Private practice<br>5 demo_setting___5 Other
6	demo_settingother<br>Show the field ONLY if:<br>[demo_setting(5)] = '1'	If other, please specify	notes<br>Custom alignment: RH
7	demo_disciplines<br>Show the field ONLY if:<br>[consent] = '1'	What disciplines provide services in your clinic?	checkbox<br>1 demo_disciplines___1 Paediatricians<br>2 demo_disciplines___2 Speech-Language Pathologists<br>3 demo_disciplines___3 Psychologists<br>4 demo_disciplines___4 Neuropsychologists<br>5 demo_disciplines___5 Occupational Therapists<br>6 demo_disciplines___6 Social Workers<br>7 demo_disciplines___7 Other
8	demo_disciplinesothers<br>Show the field ONLY if:<br>[demo_disciplines(7)] = '1'	If other, please specify	notes<br>Custom alignment: RH
9	clinic_country<br>Show the field ONLY if:<br>[consent] = '1'	What country is the clinic in?	text
10	diag_peryear<br>Show the field ONLY if:<br>[consent] = '1'	How many FASD assessments does your clinic perform per year?	text<br>Custom alignment: RH
11	waitlist<br>Show the field ONLY if:<br>[consent] = '1'	Does your clinic have a waitlist for FASD assessment?	yesno<br>1 Yes<br>0 No<br>Custom alignment: RH

12	waitlist_length<br><br>Show the field ONLY if:<br>[waitlist] = '1'	Approximately how long do clients have to wait for an assessment?	text<br>Custom alignment: RH
13	clinic_diagnostic<br><br>Show the field ONLY if:<br>[consent] = '1'	What diagnostic criteria does your clinic use to make a FASD diagnosis? Please select all the apply.	checkbox, Required<br>1 | clinic_diagnostic___1 | Australian Guide to FASD Diagnosis (Bower & Elliott, 2016)<br>2 | clinic_diagnostic___2 | Canadian Diagnostic Guideline (Chudley et al., 2005)<br>3 | clinic_diagnostic___3 | Revised Canadian Diagnostic Guideline (Cook et al., 2016)<br>4 | clinic_diagnostic___4 | United States Institute of Medicine Criteria (Hoyme et al., 2005)<br>5 | clinic_diagnostic___5 | 4-Digit Diagnostic Code (Astley, 2004)<br>6 | clinic_diagnostic___6 | Centers for Disease Control and Prevention (Fetal Alcohol Syndrome: Guidelines for Referral and Diagnosis, 2004)<br>7 | clinic_diagnostic___7 | Emory Clinic Diagnostic Guidelines<br>8 | clinic_diagnostic___8 | Other
14	diagnostic_other<br><br>Show the field ONLY if:<br>[clinic_diagnostic(8)] = '1'	If other, please specify	notes<br>Custom alignment: RH
15	diagnostic_adapt<br><br>Show the field ONLY if:<br>[consent] = '1'	Does your clinic make any adaptations to the diagnostic criteria used?	yesno<br>1 | Yes<br>0 | No<br><br>Custom alignment: RH
16	diagnostic_adapt_describe<br><br>Show the field ONLY if:<br>[diagnostic_adapt] = '1'	If yes, please describe	notes<br>Custom alignment: RH
17	domains<br><br>Show the field ONLY if:<br>[consent] = '1'	What domains are a part of your clinic's standard FASD assessment process? Please select all the apply.	checkbox, Required<br>0 | domains___0 | Prenatal Alcohol Exposure<br>1 | domains___1 | Other Prenatal Exposures or Risks<br>2 | domains___2 | Growth<br>3 | domains___3 | Facial Features<br>4 | domains___4 | Brain Structure/Neurology<br>5 | domains___5 | Motor Skills<br>6 | domains___6 | Cognition<br>7 | domains___7 | Language<br>8 | domains___8 | Academic Achievement<br>9 | domains___9 | Memory<br>10 | domains___10 | Attention<br>11 | domains___11 | Executive Function, Including Impulse control and Hyperactivity<br>12 | domains___12 | Affect Regulation<br>13 | domains___13 | Adaptive Behaviour, Social Skills or Social Communication<br>14 | domains___14 | Other

18	tools_alcoholexposure<br><br>Show the field ONLY if:<br>[domains(0)] = '1'	Section Header: *What assessment tools do you use to assess each of these domains for FASD assessments and diagnosis? Please select all that apply.*<br>Prenatal Alcohol Exposure<br><br>Please select all that apply	checkbox, Required<br>1 tools_alcoholexposure___1 Clinical Judgement<br>2 tools_alcoholexposure___2 Observation<br>3 tools_alcoholexposure___3 Interview<br>4 tools_alcoholexposure___4 Alcohol Use Disorders Identification Tests - Consumption (AUDIT-C)<br>5 tools_alcoholexposure___5 TWEAK<br>6 tools_alcoholexposure___6 T-ACE<br>7 tools_alcoholexposure___7 Alcohol, Smoking and Substance Involvement Screening Test (ASSIST)<br>0 tools_alcoholexposure___0 Other
19	tools_alcohol_other<br><br>Show the field ONLY if:<br>[tools_alcoholexposure(0)] = '1'	If other, please specify	notes
20	tools_otherexposure<br><br>Show the field ONLY if:<br>[domains(1)] = '1'	Other Prenatal Exposures and Risks<br><br>Please select all that apply	checkbox, Required<br>1 tools_otherexposure___1 Clinical Judgement<br>2 tools_otherexposure___2 Observation<br>3 tools_otherexposure___3 Interview<br>0 tools_otherexposure___0 Other
21	tools_exposure_other<br><br>Show the field ONLY if:<br>[tools_otherexposure(0)] = '1'	If other, please specify	notes
22	tools_growth<br><br>Show the field ONLY if:<br>[domains(2)] = '1'	Growth<br><br>Please select all that apply	checkbox, Required<br>1 tools_growth___1 Clinical Judgement<br>2 tools_growth___2 Observation<br>3 tools_growth___3 Birth Growth<br>4 tools_growth___4 Postnatal Growth<br>5 tools_growth___5 Parental Height<br>0 tools_growth___0 Other
23	tools_growth_other<br><br>Show the field ONLY if:<br>[tools_growth(0)] = '1'	If other, please specify	notes
24	tools_facialfeatures<br><br>Show the field ONLY if:<br>[domains(3)] = '1'	Facial Features<br><br>Please select all that apply	checkbox, Required<br>1 tools_facialfeatures___1 Clinical Judgement<br>2 tools_facialfeatures___2 Observation<br>3 tools_facialfeatures___3 University of Washington Lip-Philtrum Guide<br>4 tools_facialfeatures___4 FAS Facial Photographic Software<br>5 tools_facialfeatures___5 Palpebral Fissure Length Charts<br>6 tools_facialfeatures___6 3D Facial Photography<br>0 tools_facialfeatures___0 Other
25	tools_face_other<br><br>Show the field ONLY if:<br>[tools_facialfeatures(0)] = '1'	If other, please specify	notes

26	tools_brain_neurology<br><br>Show the field ONLY if:<br>[domains(4)] = '1'	Brain Structure/Neurology<br><br>Please select all that apply	checkbox, Required<br>1 tools_brain_neurology___1 Clinical Judgement<br>2 tools_brain_neurology___2 Observation<br>3 tools_brain_neurology___3 Birth Occipitofrontal Head Circumference (OFC)<br>4 tools_brain_neurology___4 Postnatal OFC<br>5 tools_brain_neurology___5 Evidence of seizure disorder<br>6 tools_brain_neurology___6 Evidence of other neurological diagnoses<br>7 tools_brain_neurology___7 Brain imaging to identify structural brain abnormalities<br>0 tools_brain_neurology___0 Other
27	tools_brain_other<br><br>Show the field ONLY if:<br>[tools_brain_neurology(0)] = '1'	If other, please specify	notes
28	tools_motor<br><br>Show the field ONLY if:<br>[domains(5)] = '1'	Motor Skills<br><br>Please select all that apply	checkbox, Required<br>1 tools_motor___1 Clinical Judgement<br>2 tools_motor___2 Observation<br>3 tools_motor___3 Previous non-FASD diagnoses<br>4 tools_motor___4 Bayley<br>5 tools_motor___5 Visual-Motor Integration (VMI)<br>6 tools_motor___6 Bruininks-Oseretsky Test of Motor Proficiency (BOT)<br>0 tools_motor___0 Other
29	motor_subtests_bayley<br><br>Show the field ONLY if:<br>[tools_motor(4)] = '1'	For the Bayley, please specify the subtests/screener used.	notes
30	motor_subtests_vmi<br><br>Show the field ONLY if:<br>[tools_motor(5)] = '1'	For the VMI, please specify subtests/version used if you are not administering the full battery.	notes
31	motor_subtests_bot<br><br>Show the field ONLY if:<br>[tools_motor(6)] = '1'	For the BOT, please specify subtests used if you are not administering the core battery.	notes
32	tools_motor_other<br><br>Show the field ONLY if:<br>[tools_motor(0)] = '1'	If other, please specify, including subtests where appropriate.	notes

<table>
<tr><td>33</td><td>tools_cognition

Show the field ONLY if:
[domains(6)] = '1'</td><td>Cognition

Please select all that apply</td><td>checkbox, Required
<table>
<tr><td>1</td><td>tools_cognition___1</td><td>Clinical Judgement</td></tr>
<tr><td>2</td><td>tools_cognition___2</td><td>Observation</td></tr>
<tr><td>3</td><td>tools_cognition___3</td><td>Previous non-FASD diagnoses</td></tr>
<tr><td>4</td><td>tools_cognition___4</td><td>Wechsler Intelligence Scales for Children (WISC)</td></tr>
<tr><td>5</td><td>tools_cognition___5</td><td>Bayley</td></tr>
<tr><td>6</td><td>tools_cognition___6</td><td>Differential Abilities Scales (DAS)</td></tr>
<tr><td>7</td><td>tools_cognition___7</td><td>Wechsler Preschool and Primary Scale of Intelligence (WPPSI)</td></tr>
<tr><td>8</td><td>tools_cognition___8</td><td>Stanford-Binet Intelligence Scales (SB)</td></tr>
<tr><td>9</td><td>tools_cognition___9</td><td>Wechsler Non-Verbal Scale of Ability (WNV)</td></tr>
<tr><td>10</td><td>tools_cognition___10</td><td>Wechsler Adult Intelligence Scale (WAIS)</td></tr>
<tr><td>11</td><td>tools_cognition___11</td><td>Universal Nonverbal Intelligence Test (UNIT)</td></tr>
<tr><td>12</td><td>tools_cognition___12</td><td>Naglieri Nonverbal Ability Test (NNAT)</td></tr>
<tr><td>0</td><td>tools_cognition___0</td><td>Other</td></tr>
</table></td></tr>
<tr><td>34</td><td>cognition_subtests_wisc

Show the field ONLY if:
[tools_cognition(4)] = '1'</td><td>For the WISC, please specify subtests used if not administering the core battery.</td><td>notes</td></tr>
<tr><td>35</td><td>cognition_subtests_bayley

Show the field ONLY if:
[tools_cognition(5)] = '1'</td><td>For the Bayley, please specify the subtests/screener used.</td><td>notes</td></tr>
<tr><td>36</td><td>cognition_subtests_das

Show the field ONLY if:
[tools_cognition(6)] = '1'</td><td>For the DAS, please specify subtests used if not administering the core battery.</td><td>notes</td></tr>
<tr><td>37</td><td>cognition_subtests_wppsi

Show the field ONLY if:
[tools_cognition(7)] = '1'</td><td>For the WPPSI, please specify subtests used if not administering the core battery.</td><td>notes</td></tr>
<tr><td>38</td><td>cognition_subtests_wnv

Show the field ONLY if:
[tools_cognition(9)] = '1'</td><td>For the WNV, please specify subtests used if not administering the full battery.</td><td>notes</td></tr>
<tr><td>39</td><td>cognition_subtests_wais

Show the field ONLY if:
[tools_cognition(10)] = '1'</td><td>For the WAIS, please specify subtests used if not administering the core battery.</td><td>notes</td></tr>
<tr><td>40</td><td>cognition_subtests_unit

Show the field ONLY if:
[tools_cognition(11)] = '1'</td><td>For the UNIT, please specify subtests used if not administering the core battery.</td><td>notes</td></tr>
<tr><td>41</td><td>tools_cognition_other

Show the field ONLY if:
[tools_cognition(0)] = '1'</td><td>If other, please specify, including subtests where appropriate.</td><td>notes</td></tr>
<tr><td>42</td><td>tools_language

Show the field ONLY if:
[domains(7)] = '1'</td><td>Language

Please select all that apply</td><td>checkbox, Required
<table>
<tr><td>1</td><td>tools_language___1</td><td>Clinical Judgement</td></tr>
<tr><td>2</td><td>tools_language___2</td><td>Observation</td></tr>
<tr><td>3</td><td>tools_language___3</td><td>Previous non-FASD diagnoses</td></tr>
<tr><td>4</td><td>tools_language___4</td><td>Bayley</td></tr>
<tr><td>5</td><td>tools_language___5</td><td>Pre-School Language Scales (PLS)</td></tr>
<tr><td>6</td><td>tools_language___6</td><td>Clinical Evaluation of Language Fundamentals (CELF)</td></tr>
<tr><td>0</td><td>tools_language___0</td><td>Other</td></tr>
</table></td></tr>
</table>

<table>
<tr><td>43</td><td>language_subtests_bayley
Show the field ONLY if:
[tools_language(4)] = '1'</td><td>For the Bayley, please specify the subtests/screener used.</td><td>notes</td></tr>
<tr><td>44</td><td>language_subtests_pls
Show the field ONLY if:
[tools_language(5)] = '1'</td><td>For the PLS, please specify the scales/supplementary measures used if not administering the core battery.</td><td>notes</td></tr>
<tr><td>45</td><td>language_subtests_celf
Show the field ONLY if:
[tools_language(6)] = '1'</td><td>For the CELF, please specify the subtests/screener used if not administering the core battery.</td><td>notes</td></tr>
<tr><td>46</td><td>tools_language_other
Show the field ONLY if:
[tools_language(0)] = '1'</td><td>If other, please specify, including subtests where appropriate.</td><td>notes</td></tr>
<tr><td>47</td><td>tools_academic
Show the field ONLY if:
[domains(8)] = '1'</td><td>Academic Achievement

Please select all that apply</td><td>checkbox, Required
<table>
<tr><td>1</td><td>tools_academic___1</td><td>Clinical Judgement</td></tr>
<tr><td>2</td><td>tools_academic___2</td><td>Observation</td></tr>
<tr><td>3</td><td>tools_academic___3</td><td>Previous non-FASD diagnoses</td></tr>
<tr><td>4</td><td>tools_academic___4</td><td>Teacher Rating Form (TRF)</td></tr>
<tr><td>5</td><td>tools_academic___5</td><td>School-based Standardised Literacy and Numeracy tests</td></tr>
<tr><td>6</td><td>tools_academic___6</td><td>Bracken School Readiness Assessment (BSRA)</td></tr>
<tr><td>7</td><td>tools_academic___7</td><td>Woodcock-Johnson Achievement Test (WJ)</td></tr>
<tr><td>8</td><td>tools_academic___8</td><td>Wechsler Individual Achievement Test (WIAT)</td></tr>
<tr><td>9</td><td>tools_academic___9</td><td>Wide Range Achievement Test (WRAT)</td></tr>
<tr><td>10</td><td>tools_academic___10</td><td>Kaufman Test of Educational Achievement (KTEA)</td></tr>
<tr><td>11</td><td>tools_academic___11</td><td>Peabody Individual Achievement Test (PIAT)</td></tr>
<tr><td>12</td><td>tools_academic___12</td><td>School reports with achievement levels</td></tr>
<tr><td>0</td><td>tools_academic___0</td><td>Other</td></tr>
</table></td></tr>
<tr><td>48</td><td>academic_subtests_brsa
Show the field ONLY if:
[tools_academic(6)] = '1'</td><td>For the BRSA, please specify the subtests used if not administering the full battery.</td><td>notes</td></tr>
<tr><td>49</td><td>academic_subtests_wj
Show the field ONLY if:
[tools_academic(7)] = '1'</td><td>For the WJ, please specify the battery used as well as the subtests if not administering the full battery.</td><td>notes</td></tr>
<tr><td>50</td><td>academic_subtests_wiat
Show the field ONLY if:
[tools_academic(8)] = '1'</td><td>For the WIAT, please specify the subtests used if not administering the core battery.</td><td>notes</td></tr>
<tr><td>51</td><td>academic_subtests_wrat
Show the field ONLY if:
[tools_academic(9)] = '1'</td><td>For the WRAT, please specify the subtests used if not administering the full battery.</td><td>notes</td></tr>
<tr><td>52</td><td>academic_subtests_wiat_2
Show the field ONLY if:
[tools_academic(10)] = '1'</td><td>For the KTEA please specify the subtests used if not administering the core battery.</td><td>notes</td></tr>
<tr><td>53</td><td>academic_subtests_piat
Show the field ONLY if:
[tools_academic(11)] = '1'</td><td>For the PIAT, please specify the subtests used if not administering the full battery.</td><td>notes</td></tr>
<tr><td>54</td><td>tools_academic_other
Show the field ONLY if:
[tools_academic(0)] = '1'</td><td>If other, please specify, including subtests where appropriate.</td><td>notes</td></tr>
</table>

55	tools_memory<br>Show the field ONLY if: [domains(9)] = '1'	Memory<br>Please select all that apply	checkbox, Required<br>1 tools_memory___1 Clinical Judgement<br>2 tools_memory___2 Observation<br>3 tools_memory___3 Previous non-FASD diagnoses<br>4 tools_memory___4 Differential Abilities Scales (DAS)<br>5 tools_memory___5 Behaviour Rating Inventory of Executive Function (BRIEF)<br>6 tools_memory___6 Developmental Neuropsychological Assessment (NEPSY)<br>7 tools_memory___7 Test of Problem Solving - Elementary (TOPS-E)<br>11 tools_memory___11 Test of Problem Solving - Adolescent (TOPS-A)<br>8 tools_memory___8 Wide Range Assessment of Memory and Learning (WRAML)<br>9 tools_memory___9 Wechsler Memory Scale (WMS)<br>10 tools_memory___10 Children's Memory Scale (CMS)<br>0 tools_memory___0 Other
56	memory_subtests_das<br>Show the field ONLY if: [tools_memory(4)] = '1'	For the DAS, please specify subtests used if not administering the core battery.	notes
57	memory_subtests_nepsy<br>Show the field ONLY if: [tools_memory(6)] = '1'	For the NEPSY, please specify subtests used if not administering the core battery.	notes
58	memory_subtests_topse<br>Show the field ONLY if: [tools_memory(7)] = '1'	For the TOPS-E, please specify subtests used if not administering the core battery.	notes
59	memory_subtests_topsa<br>Show the field ONLY if: [tools_memory(11)] = '1'	For the TOPS-A, please specify subtests used if not administering the core battery.	notes
60	memory_subtests_wraml<br>Show the field ONLY if: [tools_memory(8)] = '1'	For the WRAML, please specify screener/subtests used if not administering the core battery.	notes
61	memory_subtests_wms<br>Show the field ONLY if: [tools_memory(9)] = '1'	For the WMS, please specify subtests used if not administering the core battery.	notes
62	memory_subtests_wms_2<br>Show the field ONLY if: [tools_memory(10)] = '1'	For the CMS, please specify subtests used if not administering the core battery.	notes
63	tools_memory_other<br>Show the field ONLY if: [tools_memory(0)] = '1'	If other, please specify, including subtests where appropriate.	notes

64	tools_attention<br><br>Show the field ONLY if:<br>[domains(10)] = '1'	Attention<br><br>Please select all that apply	checkbox, Required<br>1 tools_attention___1 Clinical Judgement<br>2 tools_attention___2 Observation<br>3 tools_attention___3 Previous non-FASD diagnoses<br>4 tools_attention___4 Child Behaviour Checklist (CBCL)<br>5 tools_attention___5 Teacher Report Form (TRF)<br>6 tools_attention___6 Test of Everyday Attention for Children (Tea-CH)<br>15 tools_attention___15 Test of Everyday Attention (TEA)<br>7 tools_attention___7 Developmental Neuropsychological Assessment (NEPSY)<br>8 tools_attention___8 Conner's Continuous Performance Test<br>9 tools_attention___9 Delis-Kaplan Executive Function System (DKEFS)<br>10 tools_attention___10 Children's Colour Trails Test<br>11 tools_attention___11 Adult Colour Trails Test<br>12 tools_attention___12 Conners<br>13 tools_attention___13 Conners Adult ADHD Rating Scales (CAARS)<br>14 tools_attention___14 Conners Comprehensive Behaviour Rating Scales<br>0 tools_attention___0 Other
65	attention_subtests_teach<br><br>Show the field ONLY if:<br>[tools_attention(6)] = '1'	For the TEA-CH, please specify subtests used if not administering the core battery.	notes
66	attention_subtests_tea<br><br>Show the field ONLY if:<br>[tools_attention(15)] = '1'	For the TEA, please specify subtests used if not administering the core battery.	notes
67	attention_subtests_nepsy<br><br>Show the field ONLY if:<br>[tools_attention(7)] = '1'	For the NEPSY, please specify subtests used if not administering the core battery.	notes
68	attention_subtests_dkefs<br><br>Show the field ONLY if:<br>[tools_attention(9)] = '1'	For the DKEFS, please specify subtests used if not administering the core battery.	notes
69	tools_attention_other<br><br>Show the field ONLY if:<br>[tools_attention(0)] = '1'	If other, please specify, including subtests where appropriate.	notes

<table>
<tr><td>70</td><td>tools_executivefunction
Show the field ONLY if:
[domains(11)] = '1'</td><td>Executive Function, including Impulse Control and Hyperactivity
Please select all that apply</td><td>checkbox, Required<table>
<tr><td>1</td><td>tools_executivefunction___1</td><td>Clinical Judgement</td></tr>
<tr><td>2</td><td>tools_executivefunction___2</td><td>Observation</td></tr>
<tr><td>3</td><td>tools_executivefunction___3</td><td>Previous non-FASD diagnoses</td></tr>
<tr><td>4</td><td>tools_executivefunction___4</td><td>Bayley</td></tr>
<tr><td>5</td><td>tools_executivefunction___5</td><td>Executive Functions Touch</td></tr>
<tr><td>6</td><td>tools_executivefunction___6</td><td>Behaviour Rating Inventory of Executive Function (BRIEF)</td></tr>
<tr><td>7</td><td>tools_executivefunction___7</td><td>Child Behaviour Checklist (CBCL)</td></tr>
<tr><td>8</td><td>tools_executivefunction___8</td><td>Teacher Report Form (TRF)</td></tr>
<tr><td>9</td><td>tools_executivefunction___9</td><td>Cogstate</td></tr>
<tr><td>10</td><td>tools_executivefunction___10</td><td>Developmental Neuropsychological Assessment (NEPSY)</td></tr>
<tr><td>11</td><td>tools_executivefunction___11</td><td>Delis-Kaplan Executive Function System (DKEFS)</td></tr>
<tr><td>12</td><td>tools_executivefunction___12</td><td>Rey-Osterrieth Complex Figure (ROCF)</td></tr>
<tr><td>13</td><td>tools_executivefunction___13</td><td>Comprehensive Executive Function Inventory (CEFI)</td></tr>
<tr><td>14</td><td>tools_executivefunction___14</td><td>Frontal Systems Behaviour Scale (FrsBe)</td></tr>
<tr><td>15</td><td>tools_executivefunction___15</td><td>Behavior Assessment System for Children (BASC)</td></tr>
<tr><td>0</td><td>tools_executivefunction___0</td><td>Other</td></tr>
</table></td></tr>
<tr><td>71</td><td>ef_subtests_bayley
Show the field ONLY if:
[tools_executivefunction(4)] = '1'</td><td>For the Bayley, please specify the subtests/screener used.</td><td>notes</td></tr>
<tr><td>72</td><td>attention_subtests_eftouch
Show the field ONLY if:
[tools_executivefunction(5)] = '1'</td><td>For the EF Touch, please specify subtests used if not administering the full battery.</td><td>notes</td></tr>
<tr><td>73</td><td>attention_subtests_cogs
Show the field ONLY if:
[tools_executivefunction(9)] = '1'</td><td>For the Cogstate, please specify the battery used as well as the subtests if not administering the full battery.</td><td>notes</td></tr>
<tr><td>74</td><td>ef_subtests_nepsy
Show the field ONLY if:
[tools_executivefunction(10)] = '1'</td><td>For the NEPSY, please specify subtests used if not administering the core battery.</td><td>notes</td></tr>
<tr><td>75</td><td>ef_subtests_dkefs
Show the field ONLY if:
[tools_executivefunction(11)] = '1'</td><td>For the DKEFS, please specify subtests used if not administering the core battery.</td><td>notes</td></tr>
<tr><td>76</td><td>ef_subtests_basc
Show the field ONLY if:
[tools_executivefunction(15)] = '1'</td><td>For the BASC, please specify scales/forms used.</td><td>notes</td></tr>
</table>

77	tools_ef_other<br>Show the field ONLY if: [tools_executivefunction(0)] = '1'	If other, please specify, including subtests where appropriate.	notes
78	tools_affect<br>Show the field ONLY if: [domains(12)] = '1'	Affect Regulation<br><br>Please select all that apply	checkbox, Required<br>1 | tools_affect___1 | Clinical Judgement<br>2 | tools_affect___2 | Observation<br>3 | tools_affect___3 | Previous non-FASD diagnoses<br>4 | tools_affect___4 | Spence Children's Anxiety Scales (SCAS)<br>5 | tools_affect___5 | Behaviour Assessment System for Children (BASC)<br>6 | tools_affect___6 | Beck Youth Inventories<br>7 | tools_affect___7 | Children's Depression Inventory (CDI)<br>8 | tools_affect___8 | Multidimensional Anxiety Scale for Children (MASC)<br>9 | tools_affect___9 | Child Behaviour Checklist (CBCL)<br>10 | tools_affect___10 | Conners Comprehensive Behaviour Rating Scales<br>0 | tools_affect___0 | Other
79	tools_affect_other<br>Show the field ONLY if: [tools_affect(0)] = '1'	If other, please specify, including subtests where appropriate.	notes

80	tools_adaptivebeh<br>Show the field ONLY if:<br>[domains(13)] = '1'	Adaptive Behaviour, Social Skills or Social Communication<br>Please select all that apply	checkbox, Required<br>1 tools_adaptivebeh___1 Clinical Judgement<br>2 tools_adaptivebeh___2 Observation<br>3 tools_adaptivebeh___3 Previous non-FASD diagnoses<br>4 tools_adaptivebeh___4 Bayley<br>5 tools_adaptivebeh___5 Brief Infant-Toddler Social and Emotional Assessment (BITSEA)<br>6 tools_adaptivebeh___6 Child Behaviour Checklist (CBCL)<br>7 tools_adaptivebeh___7 Teacher Report Form (TRF)<br>8 tools_adaptivebeh___8 Behaviour Rating Inventory of Executive Function (BRIEF)<br>9 tools_adaptivebeh___9 Adaptive Behaviour Assessment System (ABAS)<br>10 tools_adaptivebeh___10 Social Language Development Test, Elementary (SLDT-E)<br>11 tools_adaptivebeh___11 Social Language Development Test, Adolescent (SLDT-A)<br>12 tools_adaptivebeh___12 Clinical Evaluation of Language Fundamentals (CELF)<br>13 tools_adaptivebeh___13 Vineland Adaptive Behaviour Scales (VABS)<br>14 tools_adaptivebeh___14 Behaviour Assessment System for Children (BASC)<br>15 tools_adaptivebeh___15 Pragmatic Language Observation Scale (PLOS)<br>16 tools_adaptivebeh___16 Children's Communication Checklist<br>0 tools_adaptivebeh___0 Other
81	adaptive_subtests_bayley<br>Show the field ONLY if:<br>[tools_adaptivebeh(4)] = '1'	For the Bayley, please specify the subtests/screener used.	notes
82	ef_subtests_sldt_e<br>Show the field ONLY if:<br>[tools_adaptivebeh(10)] = '1'	For the SLDT-E, please specify subtests used if not administering the full battery.	notes
83	ef_subtests_sldt_a<br>Show the field ONLY if:<br>[tools_adaptivebeh(11)] = '1'	For the SLDT-A, please specify subtests used if not administering the full battery.	notes
84	adaptive_subtest_celf<br>Show the field ONLY if:<br>[tools_adaptivebeh(12)] = '1'	For the CELF, please specify the subtests/screener used if not administering the core battery.	notes
85	adaptive_subtest_vbas<br>Show the field ONLY if:<br>[tools_adaptivebeh(13)] = '1'	For the VBAS, please specify the domains assessed if not using the full measure.	notes
86	tools_adapbeh_other<br>Show the field ONLY if:<br>[tools_adaptivebeh(0)] = '1'	If other, please specify, including subtests where appropriate.	notes
87	tools_other<br>Show the field ONLY if:<br>[domains(14)] = '1'	Other Domains (Not Listed Above)<br>Please specify domains and all tools used to assess this, including subtests where appropriate.	notes

88	assess_length<br>Show the field ONLY if:<br>[consent] = '1'	Section Header:<br>How long on average does an FASD assessment take?	text<br>Custom alignment: RH
89	assess_difficulties<br>Show the field ONLY if:<br>[consent] = '1'	Do you encounter any difficulties in your practice with the FASD assessment or diagnostic process?	yesno, Required<br>1 Yes<br>0 No<br>Custom alignment: RH
90	assess_diff_describe<br>Show the field ONLY if:<br>[assess_difficulties] = '1'	If yes, please describe	notes<br>Custom alignment: RH
91	united_diagnostic<br>Show the field ONLY if:<br>[consent] = '1'	There are multiple diagnostic criteria available around the world.<br>Do you think that it is possible to develop a unified international guideline for FASD diagnosis?	yesno, Required<br>1 Yes<br>0 No<br>Custom alignment: RH
92	united_diagnostic_worth<br>Show the field ONLY if:<br>[consent] = '1'	Do you think there is a need for a unified international guideline for FASD diagnosis?	yesno, Required<br>1 Yes<br>0 No<br>Custom alignment: RH
93	united_diag_barriers<br>Show the field ONLY if:<br>[consent] = '1'	What are the barriers to a unified international guideline for FASD diagnosis?	notes<br>Custom alignment: RH
94	united_diag_facilitators<br>Show the field ONLY if:<br>[consent] = '1'	What are the facilitators for a unified international guideline for FASD diagnosis?	notes<br>Custom alignment: RH
95	comments_suggestions<br>Show the field ONLY if:<br>[consent] = '1'	Do you have any other suggestions or comments from your experience regarding FASD assessment and diagnosis?	notes<br>Custom alignment: RH
96	diagnostic_clinic_survey_complete	Section Header: *Form Status*<br>Complete?	dropdown<br>0 Incomplete<br>1 Unverified<br>2 Complete
